# Seasonality and geographical spread of respiratory syncytial virus epidemics in 15 European countries, 2010 to 2016

**DOI:** 10.2807/1560-7917.ES.2018.23.5.17-00284

**Published:** 2018-02-01

**Authors:** Eeva K Broberg, Matti Waris, Kari Johansen, René Snacken, Pasi Penttinen, European Influenza Surveillance Network

**Affiliations:** 1 European Centre for Disease Prevention and Control (ECDC), Stockholm, Sweden; 2 University of Turku, Turku, Finland; 3 Turku University Hospital, Turku, Finland; 4 The members of the European Influenza Surveillance Network are listed at the end of the article

**Keywords:** respiratory syncytial virus, RSV, seasonality, geographic spread

## Abstract

Respiratory syncytial virus (RSV) is considered the most common pathogen causing severe lower respiratory tract infections among infants and young children. We describe the seasonality and geographical spread of RSV infection in 15 countries of the European Union and European Economic Area. We performed a retrospective descriptive study of weekly laboratory-confirmed RSV detections between weeks 40/2010 and 20/2016, in patients investigated for influenza-like illness, acute respiratory infection or following the clinician’s judgment. Six countries reported 4,230 sentinel RSV laboratory diagnoses from primary care and 14 countries reported 156,188 non-sentinel laboratory diagnoses from primary care or hospitals. The median length of the RSV season based on sentinel and non-sentinel surveillance was 16 (range: 9–24) and 18 (range: 8–24) weeks, respectively. The median peak weeks for sentinel and non-sentinel detections were week 4 (range: 48 to 11) and week 4.5 (range: 49 to 17), respectively. RSV detections peaked later (r = 0.56; p = 0.0360) and seasons lasted longer with increasing latitude (r = 0.57; p = 0.0329). Our data demonstrated regular seasonality with moderate correlation between timing of the epidemic and increasing latitude of the country. This study supports the use of RSV diagnostics within influenza or other surveillance systems to monitor RSV seasonality and geographical spread.

## Background

Respiratory syncytial virus (RSV) is considered the major pathogen causing severe lower respiratory tract infections among infants and young children [1]. RSV is the most common cause of hospitalisation for acute lower respiratory tract infection in children younger than 5 years and is estimated to cause between 66,000 and 199,000 deaths worldwide every year [2]. Its significance in causing substantial morbidity and hospitalisation in the first year of life has been affirmed in a recent study and a meta-analysis [3,4]. In England, average annual hospital admission rates are 35.1 per 1,000 children younger than 1 year and 5.31 per 1,000 children aged 1–4 years [5]. In addition to children, RSV causes a substantial disease burden in elderly people and patients with chronic obstructive pulmonary disease [6,7].

RSV causes seasonal epidemics worldwide [8], with one to two epidemics each year [9] following latitudinal gradients in timing, duration, seasonal amplitude and between-year variability [8,9]. In some studies, the seasonal periodicity has been connected to climatic factors [9-11], but a common factor that explains all observed periodicity has not been established. Meteorological conditions such as temperature and high relative humidity have been reported as important predictors of RSV epidemics [9,12]. In the United States (US) and Japan, annual national and regional variation of RSV season onset and end has been reported [13-15]. In the Nordic countries, a major outbreak often alternates with a minor one, with the minor peak in the spring and a major one the following winter [16-19], a phenomenon reported also in Croatia [20], Denmark [21] and Germany [22]. RSV antigenic groups A and B alternate in two-year cycles in Finland, with dominance of the group A viruses in years 1981–82, 1985–86 and 1989–90 and the group B viruses 1983–84 and 1987–88 [17,19], and different genotypes dominate the circulation in consecutive epidemics in Korea [23]. In Spain, no biennial rhythm has been detected but rather a stable annual epidemic with a peak between week 52 and week 1 and circulation 2–8 weeks earlier than influenza viruses [24]. Similarly, in the United Kingdom (UK), one stable epidemic per year is observed [5].

Immunoprophylaxis to prevent RSV infection with a neutralising monoclonal antibody, palivizumab, has been developed for administration to target groups on a monthly basis during the RSV season [25]. However, this drug is limited to high-risk infants, the cost prohibits its use in low- and middle-income countries and the data on effectiveness of the drug in children at high risk other than infants born at gestational age < 33 weeks and in children with chronic lung and heart diseases are limited [26]. The demonstrated high disease burden of RSV infection has created a longstanding interest in RSV vaccines. Approximately 60 RSV vaccine candidates are in preclinical to phase III clinical trials [27,28], with potential target groups including elderly people, pregnant women and infants. A vaccine is expected to enter the market within 5–10 years, presumably by 2025 [29]. As natural infection provides only limited protective immunity owing to evolution of the surface protein G and alternating dominance of antigenic groups A and B [30], most of the vaccine candidates target the fusion protein F, which is cross-reactive across RSV subtypes [27]. To circumvent issues with alternating strains, it has been also suggested to consider inclusion of both RSV A and B in a future RSV vaccine [30]. To plan optimal future vaccination strategies, it is critically important to understand who is affected by RSV and to identify which groups are at risk of more severe RSV infection requiring hospitalisation or intensive care. RSV infection is not notifiable in the European Union (EU) and European Economic Area (EEA), except in Ireland, but many countries have a long tradition of reporting laboratory-confirmed RSV infections at national and international level. The European Influenza Surveillance Network (EISN) collects RSV data for the purpose of interpreting the reports of influenza-like-illness (ILI); these data can also be used to analyse seasonality of RSV [31].

Inter-country comparative analysis of seasonal circulation of RSV across Europe is lacking as most of the published literature focuses on individual countries. Our study describes the seasonality of RSV in 15 countries in the EU/EEA, specifically the start and peak of the season, length of the season and geographical spread, as a baseline description of RSV circulation in Europe. We further aimed to test if the data reported through influenza surveillance systems in use in EU/EEA countries are appropriate to analyse RSV seasonality, including more countries and a more detailed analysis than previous studies.

## Methods

### Study design

We retrospectively studied laboratory-confirmed RSV detections reported weekly through EISN to the European Surveillance System (TESSy) hosted at the European Centre for Disease Prevention and Control (ECDC) between week 40/2010 and week 20/2016.

### Study population and data

We included reports of laboratory-confirmed RSV infection based on PCR, antigen detection, serum antibody detection or virus isolation. Clinicians used either case definitions for ILI or acute respiratory infection (ARI) as the indication for sampling, or their own judgment and diagnostic need rather than a specific case definition [32]. The specimens received from clinicians were tested for RSV in local or national laboratories, and positive results were collected through national surveillance systems. Weekly aggregated data were reported from each participating country through TESSy and covered sentinel surveillance in primary care and/or non-sentinel surveillance in primary- and/or hospital-care facilities where sampling is done for diagnostic purposes. The EU/EEA countries’ national operational contact points for influenza surveillance were consulted regarding the use of case definitions and diagnostic detection methods. The reports were dated by date of specimen collection or date of laboratory diagnosis.

Data were included if a country reported for a minimum of four seasons and 5 weeks per season, with at least 24 RSV detections per country, season and surveillance system.

To explore geographical spread of RSV infections across the EU/EEA over time, latitude and longitude of the population centre (barycentre) of each country in decimal degrees were identified. Barycentres were calculated based on the 1 km population density grid provided by Eurostat, using the 2011 population data [33], except for Iceland for which the latest available population data were from 2006.

### Data analysis

In line with similar previous work, RSV epidemic seasons were defined as the weeks when RSV detections exceeded 1.2% of total RSV-positive specimens per country, surveillance system and season [8]. RSV detections also had to exceed the threshold continuously during the season (one gap week was allowed). Sentinel and non-sentinel data were analysed separately. Average threshold values over the seasons were calculated separately per country and surveillance system.

Based on the season-specific epidemic thresholds, we calculated the median weeks in which the RSV season started and peaked as well as the median length of the seasons. The start of the season was defined as the first week when the weekly RSV detections exceeded this threshold. The season peak was defined as the week in which the maximum number of RSV detections were reported. If two weeks had the same number of detections, the first week with this number was defined as the peak week. 

The correlation between timing of the epidemic and distance of countries’ barycentre from the equator (latitude) and Greenwich meridian (longitude) was studied by applying Pearson’s correlation to the median start and peak weeks as well as the length of each RSV epidemic; this was based on data from 14 of the 15 participating countries providing non-sentinel data. Western longitudes were transformed to Eastern longitudes. Residues were tested for normal distribution by Shapiro–Wilk test. Any correlation (r) of 0–0.19 was regarded as very weak, 0.20–0.39 as weak, 0.40–0.59 as moderate, 0.60–0.79 as strong and 0.80–1 as very strong [34]. The threshold of significance was set at p = 0.05. Fitted values of the correlation, the equation of the trend line and R^2^ values (goodness of fit of the regression line) were calculated. Data were analysed with Microsoft Excel 2013 and Stata 14.

## Results

### Countries reporting RSV detections

Fifteen EU/EEA Member States reported 160,418 RSV detections during the study period: 156,188 through non-sentinel systems (14 reporting countries) and 4,230 through sentinel surveillance systems (six reporting countries) (Table). At the non-sentinel sites, four countries used the ILI case definition only, four used the ILI and ARI case definitions and six used sampling for RSV based on clinical judgement without specific case definition (Table). At the sentinel sites, two countries used the ILI case definition only and four used both ILI and ARI case definitions. Eleven countries reported non-sentinel data for all six seasons (Table). The mean number of non-sentinel detections per season ranged from 117 in Iceland to 9,026 in France. The mean number of sentinel detections per season ranged from 37 in Estonia to 322 in France.

**Table:** RSV case definitions used for sampling, geographical location and numbers of RSV detections, by country and surveillance system, 15 EU/EEA countries, 2010–2016

Country name	Case definition for sampling	Surveillance system (sentinel or non-sentinel)	Latitude of the barycentre (°North)	Longitude of the barycentre (°East)	Number of RSV seasons included	Total number of detections	Mean number of detections per season	Minimum number of detections per season	Maximum number of detections per season
Malta	ILI	Non-sentinel	35.9	14.5	5	840	168	34	264
Portugal	ILI	Non-sentinel	39.7	−9.2	4	1,305	326	79	626
Spain	Laboratory-confirmed RSV infection; testing based on clinical judgement	Non-sentinel	39.7	−3.3	6	12,706	2,118	1,278	2,965
Slovenia	ARI, ILI	Sentinel	46.2	14.9	4	191	48	41	59
France	ARI, ILI	Non-sentinel	47.1	2.7	5	45,131	9,026	8,500	9,801
	ARI, ILI	Sentinel	47.1	2.7	5	1,611	322	243	382
Germany	ILI	Non-sentinel	50.9	9.7	6	1,102	184	51	285
	ARI, ILI	Sentinel	50.9	9.7	6	1,046	174	56	336
Poland	ILI	Non-sentinel	51.7	19.3	6	1,748	291	132	464
The Netherlands	Laboratory-confirmed RSV infection; testing based on clinical judgement or ARI^a^	Non-sentinel	52.1	5.3	6	11,715	1,953	1,402	2,760
	ILI	Sentinel	52.1	5.3	5	200	40	32	50
United Kingdom	ARI, ILI	Non-sentinel	52.7	−1.6	6	50,716	8,453	4,747	10,999
	ARI, ILI	Sentinel	52.7	−1.6	6	1,033	172	63	276
Ireland	Laboratory-confirmed RSV infection; testing based on clinical judgement; notifiable since 2012^b^	Non-sentinel	53.1	−7.4	6	4,443	741	547	945
Denmark	2010/11–2014/15: ARI, ILI; 2015/16: testing based on clinical judgement^c^	Non-sentinel	55.9	10.9	6	3,006	501	45	2,568
Latvia	Laboratory-confirmed RSV infection; testing based on clinical judgement	Non-sentinel	56.8	24.4	6	2,187	365	239	442
Sweden	Laboratory-confirmed RSV infection; testing based on clinical judgement	Non-sentinel	58.9	15.4	6	18,736	3,123	1,419	5,118
Estonia	ARI, ILI	Non-sentinel	59.0	25.5	6	1,849	308	177	373
	ILI	Sentinel	59.0	25.5	4	149	37	34	48
Iceland	Laboratory-confirmed RSV infection; testing based on clinical judgement	Non-sentinel	64.4	−21.1	6	704	117	32	193
**Total**		**Non-sentinel**			**6**	**156,188**	**1,977**	**32**	**10,999**
		**Sentinel**			**6**	**4,230**	**132**	**32**	**382**

ARI: acute respiratory infection; EU/EEA: European Union/European Economic Area; GP: general practitioner; ILI: influenza-like illness; RSV: respiratory syncytial virus.

^a^ ARI used in sentinel patients; however, RSV detections reported as non-sentinel detections [38,39].

^b^ Irish case definition for notification of RSV since 2012 [40].

^c^ In seasons 2010/11 to 2014/15, all diagnostic (ARI/ILI) and sentinel specimens (ILI). In season 2015/16, all RSV-positive laboratory results from the Danish National Microbiology Database including detections from hospitals and GPs.

In Denmark, the surveillance system changed in 2015/16 from an ILI/ARI-based system to register-based retrieval of RSV-positive laboratory results from the Danish National Microbiology Database including detections from hospitals and general practitioners. Two countries, France and the UK, contributed 61% of the non-sentinel and 63% of the sentinel detections and therefore, their data influenced the overall European estimates the most.

### Seasonality

**Figure 1:**
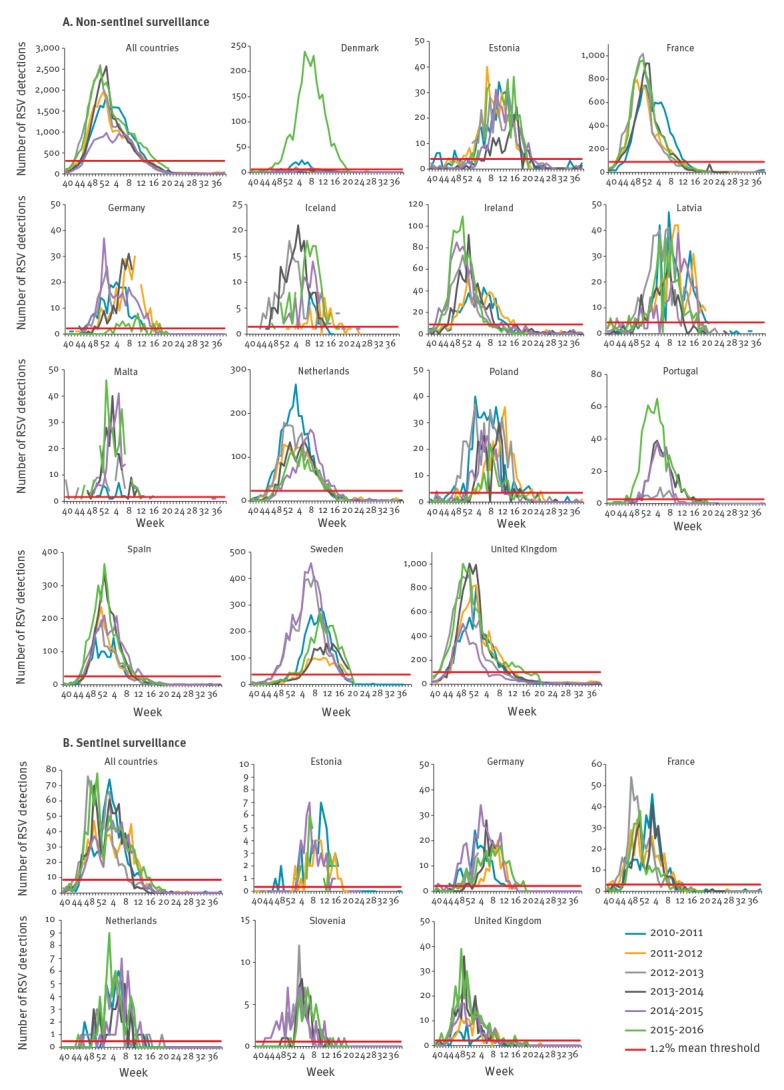
Non-sentinel (n = 14 countries) and sentinel (n = 6 countries) RSV detections by country, season and week of detection, EU/EEA, 2010–2016

Seasonality was observed by both types of surveillance (sentinel and non-sentinel) with the season threshold crossed in all countries in all years for both (Figure 1). All RSV seasons from 2010/11 to 2015/16 had a similar timing and epidemic course across Europe with some variation within and between countries (Figure 1). The highest numbers of detections were reported in seasons 2012/13, 2013/14 and 2015/16. In peak weeks, more than 2,500 specimens per week were reported positive for RSV. The lowest figures were observed in 2014/15 when France did not report RSV detections to TESSy because of a switch from one surveillance system to another during that season. Each year, the RSV epidemic in Europe progressed rapidly after week 40 to its peak and decreased to baseline levels only around week 20, which was probably driven by the later timing of the RSV epidemics in the countries with more northerly location (see below). Although the sentinel dataset was considerably smaller than the non-sentinel one, the sentinel detections followed similar patterns as non-sentinel detections and both showed a considerable effect of the end of year holiday period (weeks 52–1) (Figure 1 B) which is not as visible in the non-sentinel data (Figure 1 A). For the sentinel data, the decrease in detections during the end-of-year holiday period was mainly shown in data from Germany (Figure 1 B). While only eight detections of RSV in weeks 21–39 were reported from sentinel sources over the study period, RSV was detected by non-sentinel surveillance throughout the year, albeit at low levels (18 detections per week on average) during weeks 21–39. Compared with the remaining seasons, the season started 2–7 weeks earlier in Denmark, Germany, Iceland, Ireland, Latvia, Malta, Poland and Sweden in 2012/13 and in Germany, Poland and Sweden in 2014/15 (Figure 1 A).

EU/EEA: European Union/European Economic Area; RSV: respiratory syncytial virus.

The top left panel shows the pooled data for all included EU/EEA countries. Other panels show country-specific data by season.

**Figure 2:**
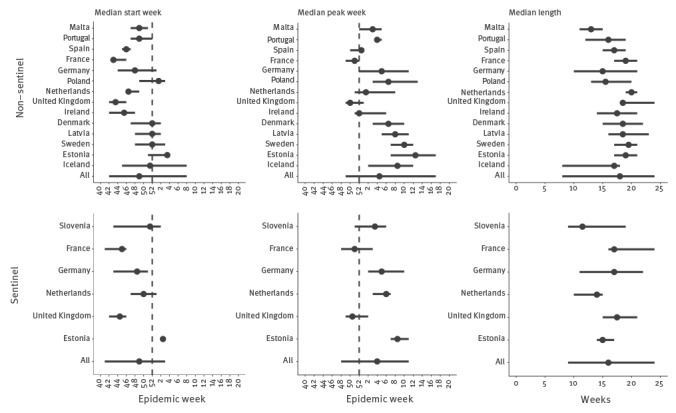
Timing of RSV season as observed by non-sentinel (n = 14 countries) and sentinel (n = 6 countries) surveillance, EU/EEA, 2010–2016

Based on analysis of the individual countries separately or as a group, the median start of the RSV season was in week 49 in both surveillance systems (ranging from week 41 to week 3 for the sentinel and from week 42 to week 8 for the non-sentinel data; Figure 2). The median peak week was in week 4 (range: 48 to 11) and 4.5 (range: 49 to 17), respectively, for sentinel and non-sentinel detections, roughly six weeks after the epidemic started. The median length of the RSV seasons based on sentinel and non-sentinel surveillance was 16 (range: 9 to 24) and 18 weeks (range: 8 to 24), respectively (Figure 2).

EU/EEA: European Union/European Economic Area; RSV: respiratory syncytial virus.

Left panels: median start week of RSV season start; middle panels: RSV season peak; Right panels: median RSV season length. Upper panels: observed by non-sentinel surveillance (14 countries); lower panels: observed by sentinel surveillance (6 countries). The dots represent the median value of a given country and the ends of the bars the minimum and maximum values of the range observed. The shift between the years is indicated by a dashed vertical line. Countries are displayed in order of increasing latitude coordinates of their barycentre. ‘All’ shows median, minimum and maximum values of the pooled results from all countries per surveillance system.

### Geographical spread

**Figure 3:**
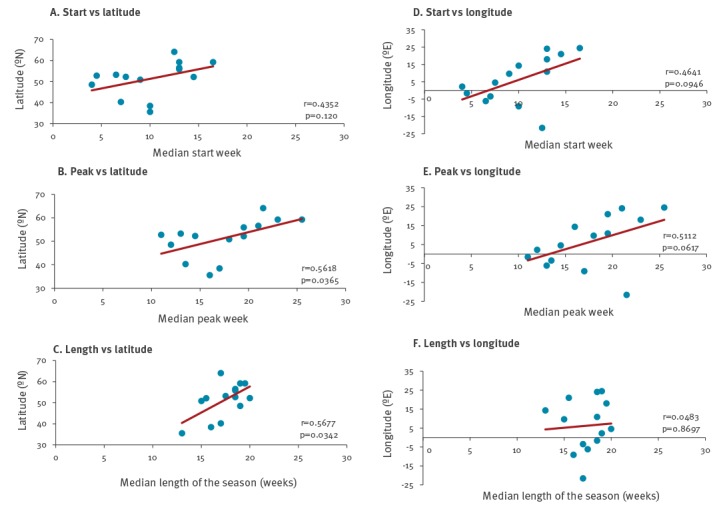
Correlation between the RSV seasonal timing and geographical location of the reporting country, non-sentinel data, EU/EEA, 2010–2016 (n = 14 countries)

RSV detections peaked later with increasing latitude (Figure 3). There was a moderate positive correlation of latitude with the peak week (r = 0.56; p = 0.0360) and the length of the season (r = 0.57; p = 0.0329). This corresponds to earlier peak and shorter seasons in the southern parts of the EU/EEA. For start and peak of the season, there were moderate correlations (r = 0.52 and r = 0.47, respectively) to increasing longitude, although without statistical significance (p = 0.0563 and p = 0.0899, respectively). 

°E: degrees east; °N: degrees north; EU/EEA: European Union/European Economic Area; RSV: respiratory syncytial virus.

The dots represent the individual data points for each country. The linear regression line of the correlation with correlation coefficient (r) and p value are indicated in each graph.

For calculation purposes, the calendar weeks were transformed into running numbers starting with 1. Calendar week 40 thus became week number 1, calendar week 41 week 2 etc. Week 53 was removed from the dataset.

## Discussion

In this study, we showed that in the 15 European countries reporting RSV surveillance data to the ECDC TESSy database, the average RSV season started in the beginning of December, peaked in early February and continued until early April, however, with wide variation between the countries. The data also showed a correlation between the earliest peak and a southerly latitude as well as between a longer season and a more northerly latitude. Furthermore, we showed that RSV seasonality can be captured through weekly reporting of RSV detections driven by diagnostic testing, using influenza ARI and ILI sentinel and non-sentinel surveillance systems. With these results, our analysis contributes to the understanding of the seasonality of RSV epidemics in Europe.

As previously shown in a global literature review and study [8], we confirmed in this study a latitudinal gradient of RSV epidemics peaking later and lasting longer with more northerly location of a country in the EU/EEA. We also showed a moderate correlation of the start and peak of the season with increasing longitude, although not statistically significant. The global study observed a weaker latitudinal gradient than our study, possibly because one third of the study sites were in the tropics [8]. In our study, all reporting countries were from the temperate climate zone in the northern hemisphere. In the global study, the northern hemisphere temperate zone covered study sites from the US to Asia, and the range for median RSV peak time was from December to February, with an epidemic duration of up to six months. The peak of the RSV epidemic in our study as well as its duration of slightly over four months is well in line with the range presented in the review [8] and in a study including seven countries from tropical and subtropical areas where the median length of a season was five months [9].

The median start week for RSV in Europe (week 49) was almost the same as for the US season which starts in week 51 (late December; range: weeks 46 to 3) [13]. The European and US epidemics both peak very close to each other: week 4 to 4.5 in our EU/EEA data and week 5 in the US [13]. Regions of the US experience significant geographical variability in the start of the RSV season corresponding to changes in longitude and latitude: in the southern states, the season starts in late November, in the Midwest in early January [13] and in Alaska between mid-October and late December [35]. In the US, an overall south-to-north gradient is usually observed for the start of the RSV epidemic [13].

The length of 13–17 weeks of the RSV season in the US [13] is a little shorter than what we observed (16–18 weeks). Interestingly, the RSV epidemics last longer in the south of the US than in the rest of the country. We observed the opposite, a statistically significant positive linear correlation between latitude and season length in the 14 studied EU/EEA countries, i.e. the further north the barycentre, the longer the RSV season. Further work is required to understand climatic and other factors which may be responsible for this correlation with latitude.

Two-year periodicity of RSV circulation has been observed in national studies in Croatia [20], Finland [17,19], Germany [22], Norway [16] and Sweden [18]. We observed an earlier start to the season only for the 2012/13 season in eight of the 15 countries and for 2014/15 in three countries. Some sporadic changes in the season start weeks and lengths were also observed.

Before the introduction of an RSV vaccine, RSV surveillance is required to document the baseline burden of disease, and the World Health Organization (WHO) is developing RSV surveillance [36] and following the RSV vaccine development with interest [29]. As no international RSV case definition has yet been agreed, the WHO has proposed candidate case definitions for severe and very severe RSV-associated lower respiratory tract infections as endpoints for RSV vaccine trials [29]. Further work is required to determine the optimal case definitions that can be applied to RSV surveillance, especially surveillance of severe outcomes. We observed that the sentinel systems detected considerably fewer RSV cases than the non-sentinel system detections, which is a reflection of the sentinel system being designed to capture only a proportion of the population under surveillance and screening mostly for influenza. The overall low numbers in sentinel systems also reflect the lower number of participating countries, and therefore lower overall population size, reporting through such a surveillance system.

Our study has a number of limitations that need to be considered when interpreting the results. Only 15 of the 30 EU/EEA Member States reported RSV detections and a large proportion originated from only two countries (France and UK), causing a skewed distribution. Therefore, results of this study should not be seen as representative for the entire EU/EEA, in particular for countries with smaller populations. No data on the patients’ ages were available and therefore analysis by age group was not possible. TESSy did not have an RSV-specific denominator of specimens tested in this data collection period, nor a population denominator for RSV, and therefore proportions of positive specimens to define season start could not be calculated and weighting factors by population size could not be used. The weighting by population size could have benefited the study by removing the strong emphasis on the data from France and the United Kingdom. Using the published method for defining the epidemic threshold [8], the epidemic threshold depends on the total number of detections per country and season and may thus be very low in a small country or during a low-intensity RSV season. We acknowledge also that only Ireland applied an RSV-specific case definition, and because the majority of detections were from non-sentinel surveillance, most of the detections were collected based on a diagnostic need. As a large proportion of the data were collected as part of the national influenza surveillance systems, efforts should be made in the future to collect specimens specifically for RSV during the influenza season as focusing mainly on influenza may have caused a bias by detecting less RSV earlier during the autumn or later in spring. The surveillance systems and detection methods were not standardised for RSV detections, many systems changed during the study period and absolute numbers of detections may therefore not be comparable across countries or within countries over time. However, thresholds were calculated by season to detect start and peak of the RSV seasons even if a small number of detections were reported. As the EU ILI case definition that is not well-suited for RSV [37] was applied in some countries, the absolute number of RSV detections may have been underestimated in these surveillance systems. Furthermore, virological data were not available and it would be of interest to study the seasonal patterns of RSV at the level of virus type and genetic clade.

Despite these limitations, the present study showed that virological surveillance systems carried out by influenza reference centres or specialist and routine diagnostic laboratories that report laboratory-confirmed RSV infections can be used to monitor RSV seasonality, confirming findings of the European Influenza Surveillance Scheme [31]. As the start of RSV monthly prophylaxis with palivizumab needs to be timed because the duration of protection is limited, according to the local circulation of the virus, knowing the seasonality at country level is of benefit for public health. At the European level, with RSV vaccines expected to come to the market in the next five to 10 years, it is crucial to establish a baseline for the number of RSV detections or for the weekly proportion of RSV-positive respiratory specimens to understand the extent of RSV circulation before implementing vaccination programmes. Further work is required to determine the design of optimal surveillance systems for RSV to measure the impact of future RSV vaccine programmes on different age groups and on the burden of severe disease. Additional benefits of establishing RSV surveillance standards at the European level include the ability to compare seasonality and trends between countries as well as virus typing and genetic characterisation, and to obtain standardised data on age groups at risk, such as newborns.

## Notes

**European Influenza Surveillance Network members from the contributing countries:** Denmark: Ramona Trebbien, Hanne-Dorthe Emborg, Tyra Grove Krause and Thea K Fischer (Statens Serum Institut, Copenhagen). Estonia: Natalja Kuznetsova, Liidia Dotsenko (Estonian Health Board, Laboratory of Communicable Diseases, Tallinn) and Olga Sadikova (Estonian Health Board, Department of CD surveillance and control, Influenza Centre, Tallinn). France: Sylvie Behillil (Unité de Génétique Moléculaire des Virus à ARN, UMR 3569 CNRS, Université Paris Diderot SPC, Institut Pasteur, Paris; CNR des Virus Influenzae, Institut Pasteur, Paris), Thierry Blanchon (Sorbonne Université, INSERM, Institut Pierre Louis d’Epidémiologie et de Santé Publique (IPLESP), F75012 Paris, France), Lisandru Capai (Sorbonne Université, INSERM, Institut Pierre Louis d’Epidémiologie et de Santé Publique (IPLESP), F75012 Paris, France), F75012, Paris, ; EA7310, Laboratoire de Virologie, Université de Corse-Inserm, Corte), Jean-Marie Cohen (Open Rome (Organize and Promote Epidemiological Network), Paris, France, Réseau des GROG, Paris), Vincent Enouf (Unité de Génétique Moléculaire des Virus à ARN, UMR 3569 CNRS, Université Paris Diderot SPC, Institut Pasteur, Paris; CNR des Virus Influenzae, Institut Pasteur, Paris), Alessandra Falchi (EA7310, Laboratoire de Virologie, Université de Corse-Inserm, Corte), Caroline Guerrisi (Sorbonne Université, INSERM, Institut Pierre Louis d’Epidémiologie et de Santé Publique (IPLESP), F75012 Paris, France), Bruno Lina (Laboratoire de Virologie, CNR des virus influenza, Institut des Agents Infectieux, Groupement Hospitalier Nord des HCL, Lyon, Laboratoire Virpath, CIRI Inserm U1111, CNRS 5308, ENS, UCBL, Faculté de Médecine LYON Est, Université de Lyon, Lyon), Thomas Hanslik (AP-HP, Hôpital Ambroise Paré, Service de Médecine interne, F-92100 Boulogne Billancourt, France), Shirley Masse (Sorbonne Université, INSERM, Institut Pierre Louis d’Epidémiologie et de Santé Publique (IPLESP), F75012 Paris, France; EA7310, Laboratoire de Virologie, Université de Corse-Inserm, Corte), Anne Mosnier (Open Rome (Organize and Promote Epidemiological Network), Paris, France; Réseau des GROG, Paris), Clément Turbelin (Sorbonne Université, INSERM, Institut Pierre Louis d’Epidémiologie et de Santé Publique (IPLESP), F75012 Paris, France), Martine Valette (Laboratoire de Virologie, CNR des virus influenza, Institut des Agents Infectieux, Groupement Hospitalier Nord des HCL, Lyon, Laboratoire Virpath, CIRI Inserm U1111, CNRS 5308, ENS, UCBL, Faculté de Médecine LYON Est, Université de Lyon, Lyon), Sylvie van der Werf (Unité de Génétique Moléculaire des Virus à ARN, UMR 3569 CNRS, Université Paris Diderot SPC, Institut Pasteur, Paris; CNR des Virus Influenzae, Institut Pasteur, Paris). Germany: Brunhilde Schweiger, Janine Reiche, Barbara Biere (National Influenza Centre, Robert Koch Institute, Berlin) and Silke Buda (Respiratory Infections Unit, Department for Infectious Disease Epidemiology, Robert Koch Institute, Berlin). Iceland: Gudrun Erna Baldvinsdottir (Department of Clinical Microbiology at Landspitali University Hospital, Reykjavik). Ireland: Lisa Domegan and Joan O Donnell (HSE-Health Protection Surveillance Centre, Dublin), Christina Byrne, Paul O’Reilly, Joanne Moran, Allison Waters and Cillian de Gascun (National Virus Reference Laboratory, University College Dublin, Dublin). Latvia: Natalija Zamjatina and Gatis Pakarna (Riga East University Hospital, Latvian Centre of Infectious Diseases, Riga) and Raina Nikiforova (Centre for Disease Prevention and Control, Riga). Malta: Jackie Maistre Melillo and Tanya Melillo (Infectious Disease Prevention and Control Unit, Msida). The Netherlands: Adam Meijer (Centre for Infectious Diseases Research, Diagnostics and Laboratory Surveillance, National Institute for Public Health and the Environment (RIVM), Bilthoven), Anne Teirlinck (Centre for Infectious Diseases, Epidemiology and Surveillance, RIVM) and Gé Donker (NIVEL, Netherlands Institute for Health Services Research, Utrecht). Poland: Lidia B. Brydak and Katarzyna Cieślak (National Influenza Centre, Department of Influenza Research, National Institute of Public Health - National Institute of Hygiene, Warsaw). Portugal: Raquel Guiomar, Paula Cristóvão and Pedro Pechirra (National Influenza Reference Laboratory, Infectious Diseases Department, National Institute of Health Dr Ricardo Jorge, I.P., Lisbon) and Ana Paula Rodrigues (Department of Epidemiology, National Institute of Health Dr Ricardo Jorge, I.P., Lisbon). Slovenia: Katarina Prosenc (National Laboratory for Health, Environment and Food, Slovenia) and Maja Socan (Public Health Institute, Slovenia). Spain: Amparo Larrauri, Concepcion Delgado-Sanz, Jesús Oliva, (National Centre of Epidemiology, CIBERESP, Instituto de Salud Carlos III, Madrid); Silvia Jiménez-Jorge (National Centre of Epidemiology, Instituto de Salud Carlos III, Madrid), Francisco Pozo, Inmaculada Casas (National Centre of Microbiology, Instituto de Salud Carlos III, Madrid); Jose María Navarro (Hospital Virgen de las Nieves de Granada); Virtudes Gallardo (Consejería de Salud, Andalucía); Manuel Omeñaca (HU Miguel Servet); Elisa Marco (Dirección General de Salud Pública, Aragón); Santiago Melón (HU Central de Asturias); Ismael Huerta (Dirección General de Salud Pública, Asturias); Jordi Reina (Hospital Son Espases de Palma de Mallorca); Carmen Pérez González (Hospital Dr Negrín de Las Palmas de Gran Canarias); Lucas González (Dirección General de Salud Pública, Canarias); Microbiologists of the Hospital Network of Castilla La Mancha; Maria Victoria García Rivera (Servicio de Epidemiología, Consejería de Sanidad); Raúl Ortiz de Lejarazu (National Influenza Center, Hospital Clínico Universitario Valladolid); Tomás Vega (Observatorio de Salud Pública, Castilla y León); Mª Ángeles Marcos (Hospital Clínico de Barcelona); Nuria Torner (Agència de Salut Pública, CIBERESP, Cataluña); Sonia Perez Castro (CH Meixoeiro, Vigo); Juan Garcia Costa (CH Santa María Nai, Ourense); María Jesús Purriños (Dirección Xeral Saúde Pública, Galicia); Antonio Moreno Docón (Hospital Virgen de la Arrixaca); Miriam Fernández (Clínica Universitaria de Navarra); Carmen Ezpeleta (Complejo Hospitalario de Navarra); Jesús Castilla (Instituto de Salud Pública, CIBERESP; Navarra); Miriam Blasco (Hospital de La Rioja de Logroño); Carmen Quiñones (Dirección General de Salud Pública y Consumo, La Rioja); José López Barba (Hospital de INGESA); Ana Rivas (Consejería de Sanidad y Bienestar Social, Ceuta). Sweden: AnnaSara Carnahan, Sarah Axelsson, Hélène Englund, Marie Rapp and Mia Brytting (Public Health Agency of Sweden, Stockholm). United Kingdom: Richard Pebody (PHE, Colindale); Joanna Ellis (PHE, Colindale); Maria Zambon (PHE, Colindale), Simon de Lusignan (Royal College of General Practitioners, London), Naomh Gallagher, Chris Nugent and Cathriona Kearns (Public Health Agency, Northern Ireland), Simon Cottrell and Catherine Moore (Public Health Wales, Cardiff), Jim McMenamin (HPS, Glasgow).

## References

[1] Simoes EA Respiratory syncytial virus infection. Lancet. 1999;354(9181):847-52. 10.1016/S0140-6736(99)80040-3 10485741

[2] Nair H Nokes DJ Gessner BD Dherani M Madhi SA Singleton RJ Global burden of acute lower respiratory infections due to respiratory syncytial virus in young children: a systematic review and meta-analysis. Lancet. 2010;375(9725):1545-55. 10.1016/S0140-6736(10)60206-1 20399493 PMC2864404

[3] Heikkinen T Ojala E Waris M Clinical and socioeconomic burden of respiratory syncytial virus infection in children. J Infect Dis. 2017;215(1):17-23. 10.1093/infdis/jiw475 27738052

[4] Stein RT Bont LJ Zar H Polack FP Park C Claxton A Respiratory syncytial virus hospitalization and mortality: Systematic review and meta-analysis. Pediatr Pulmonol. 2017;52(4):556-69. 10.1002/ppul.23570 27740723 PMC5396299

[5] Reeves RM Hardelid P Gilbert R Warburton F Ellis J Pebody RG Estimating the burden of respiratory syncytial virus (RSV) on respiratory hospital admissions in children less than five years of age in England, 2007-2012. Influenza Other Respi Viruses. 2017;11(2):122-9. 10.1111/irv.12443 28058797 PMC5304572

[6] Falsey AR Hennessey PA Formica MA Cox C Walsh EE Respiratory syncytial virus infection in elderly and high-risk adults. N Engl J Med. 2005;352(17):1749-59. 10.1056/NEJMoa043951 15858184

[7] Zwaans WA Mallia P van Winden ME Rohde GG The relevance of respiratory viral infections in the exacerbations of chronic obstructive pulmonary disease—a systematic review. J Clin Virol. 2014;61(2):181-8. 10.1016/j.jcv.2014.06.025 25066886 PMC7106508

[8] Bloom-Feshbach K Alonso WJ Charu V Tamerius J Simonsen L Miller MA Latitudinal variations in seasonal activity of influenza and respiratory syncytial virus (RSV): a global comparative review. PLoS One. 2013;8(2):e54445. 10.1371/journal.pone.0054445 23457451 PMC3573019

[9] Haynes AK Manangan AP Iwane MK Sturm-Ramirez K Homaira N Brooks WA Respiratory syncytial virus circulation in seven countries with Global Disease Detection Regional Centers. J Infect Dis. 2013;208(Suppl 3):S246-54. 10.1093/infdis/jit515 24265484

[10] Stensballe LG Devasundaram JK Simoes EA Respiratory syncytial virus epidemics: the ups and downs of a seasonal virus. Pediatr Infect Dis J. 2003;22(2) Suppl;S21-32. 10.1097/00006454-200302001-00004 12671449

[11] Yusuf S Piedimonte G Auais A Demmler G Krishnan S Van Caeseele P The relationship of meteorological conditions to the epidemic activity of respiratory syncytial virus. Epidemiol Infect. 2007;135(7):1077-90. 10.1017/S095026880600776X 17346359 PMC2870672

[12] Meerhoff TJ Paget JW Kimpen JL Schellevis F Variation of respiratory syncytial virus and the relation with meteorological factors in different winter seasons. Pediatr Infect Dis J. 2009;28(10):860-6. 10.1097/INF.0b013e3181a3e949 20118684

[13] Mullins JA Lamonte AC Bresee JS Anderson LJ Substantial variability in community respiratory syncytial virus season timing. Pediatr Infect Dis J. 2003;22(10):857-62. 10.1097/01.inf.0000090921.21313.d3 14551484

[14] Mizuta K Abiko C Aoki Y Ikeda T Matsuzaki Y Itagaki T Seasonal patterns of respiratory syncytial virus, influenza A virus, human metapneumovirus, and parainfluenza virus type 3 infections on the basis of virus isolation data between 2004 and 2011 in Yamagata, Japan. Jpn J Infect Dis. 2013;66(2):140-5. 10.7883/yoken.66.140 23514911

[15] Haynes AK Prill MM Iwane MK Gerber SI Centers for Disease Control and Prevention (CDC) Respiratory syncytial virus--United States, July 2012-June 2014. MMWR Morb Mortal Wkly Rep. 2014;63(48):1133-6. 25474034 PMC4584603

[16] Anestad G Surveillance of respiratory viral infections by rapid immunofluorescence diagnosis, with emphasis on virus interference. Epidemiol Infect. 1987;99(2):523-31. 10.1017/S0950268800068023 2824225 PMC2249295

[17] Waris M Pattern of respiratory syncytial virus epidemics in Finland: two-year cycles with alternating prevalence of groups A and B. J Infect Dis. 1991;163(3):464-9. 10.1093/infdis/163.3.464 1995719

[18] Eriksson M Bennet R Rotzén-Ostlund M von Sydow M Wirgart BZ Population-based rates of severe respiratory syncytial virus infection in children with and without risk factors, and outcome in a tertiary care setting. Acta Paediatr. 2002;91(5):593-8. 10.1111/j.1651-2227.2002.tb03282.x 12113331

[19] White LJ Waris M Cane PA Nokes DJ Medley GF The transmission dynamics of groups A and B human respiratory syncytial virus (hRSV) in England & Wales and Finland: seasonality and cross-protection. Epidemiol Infect. 2005;133(2):279-89. 10.1017/S0950268804003450 15816153 PMC2870247

[20] Mlinaric-Galinovic G Welliver RC Vilibic-Cavlek T Ljubin-Sternak S Drazenovic V Galinovic I The biennial cycle of respiratory syncytial virus outbreaks in Croatia. Virol J. 2008;5(1):18. 10.1186/1743-422X-5-18 18226194 PMC2267449

[21] Jepsen MT Trebbien R Emborg HD Krause TG Schønning K Voldstedlund M Incidence and seasonality of respiratory syncytial virus hospitalisations in young children in Denmark, 2010 to 2015. Euro Surveill. 2018;23(3):17-00163. 10.2807/1560-7917.ES.2018.23.3.17-00163 PMC5792699 29386093

[22] Terletskaia-Ladwig E Enders G Schalasta G Enders M Defining the timing of respiratory syncytial virus (RSV) outbreaks: an epidemiological study. BMC Infect Dis. 2005;5(1):20. 10.1186/1471-2334-5-20 15801975 PMC1084247

[23] Choi EH Lee HJ Genetic diversity and molecular epidemiology of the G protein of subgroups A and B of respiratory syncytial viruses isolated over 9 consecutive epidemics in Korea. J Infect Dis. 2000;181(5):1547-56. 10.1086/315468 10823752

[24] Jiménez-Jorge S Delgado-Sanz C de Mateo S Pozo F Casas I Larrauri A Sistema de Vigilancia de Gripe en España (SVGE) [Monitoring respiratory syncytial virus through the Spanish influenza surveillance system, 2006-2014]. Enferm Infecc Microbiol Clin. 2016;34(2):117-20. 10.1016/j.eimc.2014.12.012 25703209

[25] Groothuis JR Hoopes JM Hemming VG Prevention of serious respiratory syncytial virus-related illness. II: Immunoprophylaxis. Adv Ther. 2011;28(2):110-25. 10.1007/s12325-010-0101-y 21318605

[26] Homaira N, Rawlinson W, Snelling TL, Jaffe A. Effectiveness of palivizumab in preventing RSV hospitalization in high risk children: a real-world perspective. Int J Pediatr. 2014;2014:571609. 10.1155/2014/571609 PMC4274815 25548575

[27] Higgins D Trujillo C Keech C Advances in RSV vaccine research and development - A global agenda. Vaccine. 2016;34(26):2870-5. 10.1016/j.vaccine.2016.03.109 27105562

[28] Roberts JN Graham BS Karron RA Munoz FM Falsey AR Anderson LJ Challenges and opportunities in RSV vaccine development: Meeting report from FDA/NIH workshop. Vaccine. 2016;34(41):4843-9. 10.1016/j.vaccine.2016.07.057 27566900

[29] Modjarrad K Giersing B Kaslow DC Smith PG Moorthy VS WHO RSV Vaccine Consultation Expert Group WHO consultation on respiratory syncytial virus vaccine development. Report from a World Health Organization meeting held on 23-24 March 2015. Vaccine. 2016;34(2):190-7. 10.1016/j.vaccine.2015.05.093 26100926 PMC6858870

[30] Melero JA Moore ML Influence of respiratory syncytial virus strain differences on pathogenesis and immunity. Curr Top Microbiol Immunol. 2013;372:59-82. 10.1007/978-3-642-38919-1_3 24362684 PMC4880365

[31] Meerhoff TJ Mosnier A Schellevis F Paget WJ EISS RSV Task Group Progress in the surveillance of respiratory syncytial virus (RSV) in Europe: 2001-2008. Euro Surveill. 2009;14(40):19346. 19822120

[32] European Commission. Commission Decision of 28 April 2008 amending Decision 2002/253/EC laying down case definitions for reporting communicable diseases to the Community network under Decision No 2119/98/EC of the European Parliament and of the Council. 2008/426/EC. Official Journal of the European Union. 2008; L 159/46. Available from: https://publications.europa.eu/en/publication-detail/-/publication/3e53de24-26d6-4645-b9ab-3931f3874c9e/language-en

[33] Eurostat. GEOSTAT 1 km^2^ population grid, period 2011, version date 01/02/2016. Luxembourg: European Commission. [Accessed: 29 Mar 2017]. Available from: http://ec.europa.eu/eurostat/web/gisco/geodata/reference-data/population-distribution-demography/geostat

[34] Swinscow TDV. Correlation and regression 2016 In: Statistics at square one. 9th ed. London: BMJ Publishing Group Ltd; 1997. Available from: http://www.bmj.com/about-bmj/resources-readers/publications/statistics-square-one/11-correlation-and-regression

[35] Bruden DJ Singleton R Hawk CS Bulkow LR Bentley S Anderson LJ Eighteen years of respiratory syncytial virus surveillance: changes in seasonality and hospitalization rates in southwestern Alaska native children. Pediatr Infect Dis J. 2015;34(9):945-50. 10.1097/INF.0000000000000772 26065863 PMC6931377

[36] World Health Organization (WHO). WHO informal consultation on surveillance of RSV on the Global Influenza Surveillance and Response System (GISRS) platform 2015. Meeting report. Geneva: WHO; 2017. Available from: http://www.who.int/influenza/resources/publications/report_rsv_meeting/en/

[37] Iwane MK Farnon EC Gerber SI Importance of global surveillance for respiratory syncytial virus. J Infect Dis. 2013;208(Suppl 3):S165-6. 10.1093/infdis/jit484 24265473

[38] Donker GA. NIVEL primary care databases - sentinel practices. ISBN/EAN 9789461223791. Utrecht: Nederlands instituut voor onderzoek van de gezondheidszorg; 2016. Available from: http://www.nivel.nl/node/2430?database=ChoicePublicat&priref=1003052

[39] Rijksinstituut voor Voksgezondheid en Milieu (RIVM). Virologische weekstaten. [Weekly virological data [Accessed: 29 Mar 2017]. Dutch. Available from: http://www.rivm.nl/Onderwerpen/V/Virologische_weekstaten

[40] Health Protection Surveillance Centre (HPSC). Case definitions for notifiable diseases. 2012 Version 1.8. Dublin: HPSC; 2012. Available from: http://www.hpsc.ie/NotifiableDiseases/CaseDefinitions/File,823,en.pdf

